# Ethyl­triphenyl­phospho­nium bromide dihydrate

**DOI:** 10.1107/S1600536811026559

**Published:** 2011-07-09

**Authors:** Richard Betz, Thomas Gerber

**Affiliations:** a Nelson Mandela Metropolitan University, Summerstrand Campus, Department of Chemistry, University Way, Summerstrand, PO Box 77000, Port Elizabeth 6031, South Africa

## Abstract

In the crystal structure of the title hydrated bromide salt, C_20_H_20_P^+^·Br^−^·2H_2_O, O—H⋯Br and O—H⋯O hydrogen bonds as well as C—H⋯Br contacts connect the different components into a three-dimensional network. In the cation, the aromatic rings make dihedral angles of 55.24 (5), 76.16 (4) and 85.68 (4)°.

## Related literature

For the crystal structures of the monohydrate as well as the dihydrate of tetra­phenyl­phospho­nium bromide, see: Vincent *et al.* (1988 ▶); Krug & Müller (1990 ▶). For graph-set analysis of hydrogen bonds, see: Etter *et al.* (1990 ▶); Bernstein *et al.* (1995 ▶). 
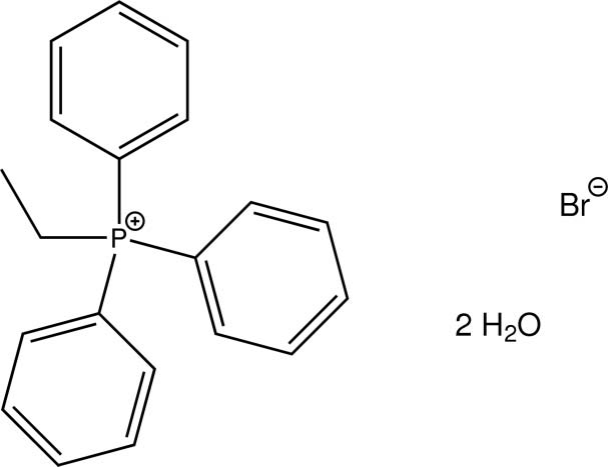


## Experimental

### 

#### Crystal data

C_20_H_20_P^+^·Br^−^·2H_2_O
*M* *_r_* = 407.27
Orthorhombic, 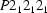

*a* = 8.8030 (2) Å
*b* = 12.7450 (3) Å
*c* = 17.5779 (3) Å
*V* = 1972.14 (7) Å^3^
*Z* = 4
Mo *K*α radiation
μ = 2.17 mm^−1^
*T* = 200 K
0.50 × 0.43 × 0.26 mm

#### Data collection

Bruker APEXII CCD diffractometer
Absorption correction: multi-scan (*SADABS*; Bruker, 2008 ▶) *T*_min_ = 0.750, *T*_max_ = 1.000
17777 measured reflections
4847 independent reflections
4616 reflections with *I* > 2σ(*I*)
*R*_int_ = 0.015

#### Refinement

*R*[*F* ^2^ > 2σ(*F* ^2^)] = 0.018
*wR*(*F* ^2^) = 0.048
*S* = 1.04
4847 reflections
235 parameters
6 restraints
H atoms treated by a mixture of independent and constrained refinement
Δρ_max_ = 0.36 e Å^−3^
Δρ_min_ = −0.25 e Å^−3^
Absolute structure: Flack (1983 ▶), 2057 Friedel pairs
Flack parameter: 0.001 (4)

### 

Data collection: *APEX2* (Bruker, 2010 ▶); cell refinement: *SAINT* (Bruker, 2010 ▶); data reduction: *SAINT*; program(s) used to solve structure: *SHELXS97* (Sheldrick, 2008 ▶); program(s) used to refine structure: *SHELXL97* (Sheldrick, 2008 ▶); molecular graphics: *ORTEP-3 for Windows* (Farrugia, 1997 ▶) and *Mercury* (Macrae *et al.*, 2008 ▶); software used to prepare material for publication: *SHELXL97* and *PLATON* (Spek, 2009 ▶).

## Supplementary Material

Crystal structure: contains datablock(s) I, global. DOI: 10.1107/S1600536811026559/ez2252sup1.cif

Supplementary material file. DOI: 10.1107/S1600536811026559/ez2252Isup2.cdx

Structure factors: contains datablock(s) I. DOI: 10.1107/S1600536811026559/ez2252Isup3.hkl

Supplementary material file. DOI: 10.1107/S1600536811026559/ez2252Isup4.cml

Additional supplementary materials:  crystallographic information; 3D view; checkCIF report

## Figures and Tables

**Table 1:** Hydrogen-bond geometry (Å, °)

*D*—H⋯*A*	*D*—H	H⋯*A*	*D*⋯*A*	*D*—H⋯*A*
O90—H901⋯Br1^i^	0.83 (2)	2.71 (2)	3.5137 (18)	162 (2)
O90—H902⋯Br1	0.84 (2)	2.57 (2)	3.3806 (15)	163 (3)
O91—H911⋯O90^i^	0.83 (2)	2.10 (2)	2.869 (3)	155 (3)
O91—H912⋯Br1	0.84 (2)	2.70 (2)	3.5315 (17)	174 (3)
C35—H35⋯Br1^ii^	0.95	2.95	3.8305 (16)	155
C1—H1*B*⋯Br1^iii^	0.99	2.70	3.6843 (14)	174

Symmetry codes: (i) 
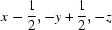
; (ii) 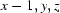; (iii) 
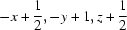
.

**Fig. 1.**
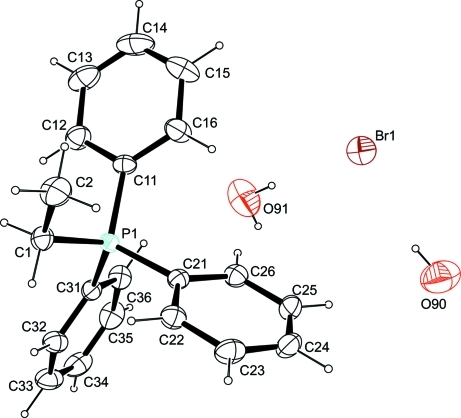
The molecular structure of the title compound, with atom labels and anisotropic displacement ellipsoids (drawn at 50% probability level).

**Fig. 2.**
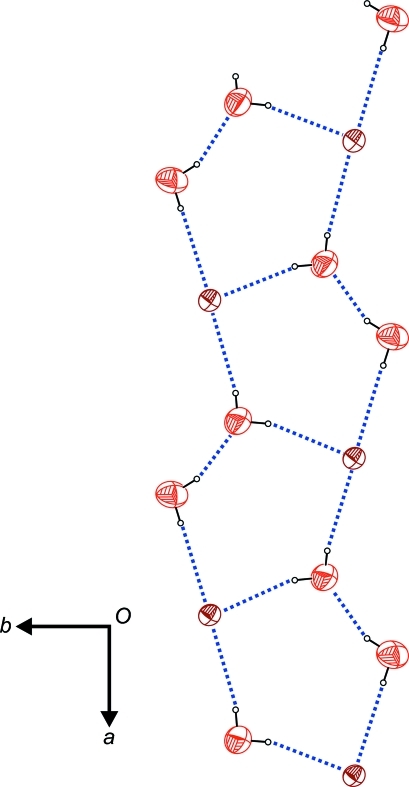
Intermolecular contacts, indicated by dashed lines, viewed along [0 0 - 1]. For clarity, only the water molecules as well as the bromide anion are depicted.

**Fig. 3.**
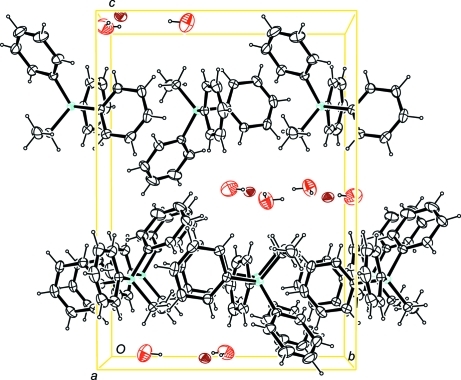
Molecular packing of the title compound, viewed along [-1 0 0] (anisotropic displacement ellipsoids drawn at 50% probability level).

## supplementary crystallographic information

### Comment

The crystallization of ionic compounds is strongly influenced by the relative spatial size ratio of anion to cation, and the presence of different anions may influence the conformation and as well as metric parameters of the cation in the case of bigger, organic cations. At the beginning of a comprehensive study of the influence of various anions on bond lengths and angles among a series of tetra-organo phosphonium compounds, we determined the molecular and crystal structure of the title compound. The molecular structure of the monohydrate as well as the dihydrate of tetraphenylphosphonium bromide are apparent in the literature (Vincent *et al.*, 1988; Krug & Müller, 1990).

The molecular geometry around the P atom is tetrahedral with the respective C–P–C angles covering a range of 105.57 (5)–112.48 (6) °, where the biggest as well as the smallest angle are enclosed between two phenyl groups. The least-squares planes defined by the carbon atoms of the aromatic moieties intersect at angles of 55.24 (5) °, 76.16 (4) ° and 85.68 (4) °. The methyl group of the ethyl substituent adopts a staggered conformation with respect to two of the three phenyl groups if the cation is projected along the phosphorus–ethyl bond.

In the molecule, hydrogen bonds are apparent between the water molecules as well as the bromide anion. The latter also serves as acceptor for C–H···Br contacts that stem from one of the H atoms on a phenyl group's *meta*-position as well as one of the H atoms of the ethyl substituent's CH_2_ group. While the first of these C–H···Br contacts falls only by about 0.1 Å below the sum of van der Waals radii, the latter one shows a shortening by more than 0.3 Å. In terms of graph-set analysis (Etter *et al.*, 1990; Bernstein *et al.*, 1995), the descriptor for the hydrogen bonds is *DDDD* on the unitary level, while the C–H···Br contacts necessitate a *DD* descriptor on the same level. In total, the components of the crystal structure are connected to form a three-dimensional network with the water molecules and bromide anions forming strands along the crystallographic *a* axis (Fig. 2). The closest intercentroid distance between two π-systems was measured at 4.5109 (7) Å.

The packing of the title compound is shown in Figure 3.

### Experimental

The compound was obtained commercially (KEK). Crystals suitable for the X-ray diffraction study were obtained upon recrystallization from boiling water with subsequent evaporation of the solvent at ambient temperature.

### Refinement

Carbon-bound H atoms were placed in calculated positions (C—H 0.95 Å for aromatic C atoms and C—H 0.99 Å for methylene groups) and were included in the refinement in the riding model approximation, with *U*(H) set to 1.2*U*_eq_(C). The hydrogen atoms of the methyl group were allowed to rotate with a fixed angle around the C–C bond to best fit the experimental electron density [HFIX 137 in the *SHELX* program suite (Sheldrick, 2008)]. The H atoms of the water molecules were located on a difference Fourier map and refined using a *DFIX* instruction (*d*_O—H_ set to 0.83 Å), with individual thermal parameters.

### Crystal data

**Table:**

C_20_H_20_P^+^·Br^−^·2H_2_O	*F*(000) = 840
*M**_r_* = 407.27	*D*_x_ = 1.372 Mg m^−^^3^
Orthorhombic, *P*2_1_2_1_2_1_	Mo *K*α radiation, λ = 0.71073 Å
Hall symbol: P 2ac 2ab	Cell parameters from 9915 reflections
*a* = 8.8030 (2) Å	θ = 2.6–28.3°
*b* = 12.7450 (3) Å	µ = 2.17 mm^−^^1^
*c* = 17.5779 (3) Å	*T* = 200 K
*V* = 1972.14 (7) Å^3^	Block, colourless
*Z* = 4	0.50 × 0.43 × 0.26 mm

### Data collection

**Table:**

Bruker APEXII CCD diffractometer	4847 independent reflections
Radiation source: fine-focus sealed tube	4616 reflections with *I* > 2σ(*I*)
graphite	*R*_int_ = 0.015
φ and ω scans	θ_max_ = 28.3°, θ_min_ = 2.0°
Absorption correction: multi-scan (*SADABS*; Bruker, 2008)	*h* = −11→11
*T*_min_ = 0.750, *T*_max_ = 1.000	*k* = −17→17
17777 measured reflections	*l* = −23→23

### Refinement

**Table:**

Refinement on *F*^2^	Secondary atom site location: difference Fourier map
Least-squares matrix: full	Hydrogen site location: inferred from neighbouring sites
*R*[*F*^2^ > 2σ(*F*^2^)] = 0.018	H atoms treated by a mixture of independent and constrained refinement
*wR*(*F*^2^) = 0.048	*w* = 1/[σ^2^(*F*_o_^2^) + (0.0125*P*)^2^ + 0.040*P*] where *P* = (*F*_o_^2^ + 2*F*_c_^2^)/3
*S* = 1.04	(Δ/σ)_max_ = 0.001
4847 reflections	Δρ_max_ = 0.36 e Å^−^^3^
235 parameters	Δρ_min_ = −0.25 e Å^−^^3^
6 restraints	Absolute structure: Flack (1983), 2057 Friedel pairs
Primary atom site location: structure-invariant direct methods	Flack parameter: 0.001 (4)

### Fractional atomic coordinates and isotropic or equivalent isotropic displacement parameters (Å^2^)

**Table:**

	*x*	*y*	*z*	*U*_iso_*/*U*_eq_	
P1	0.11207 (4)	0.62436 (2)	0.247940 (19)	0.01803 (6)	
C1	0.14585 (17)	0.71121 (9)	0.32676 (8)	0.0254 (3)	
H1A	0.0827	0.7749	0.3207	0.031*	
H1B	0.1148	0.6761	0.3745	0.031*	
C2	0.31276 (19)	0.74301 (11)	0.33249 (10)	0.0370 (4)	
H2A	0.3430	0.7799	0.2860	0.056*	
H2B	0.3756	0.6801	0.3388	0.056*	
H2C	0.3268	0.7894	0.3764	0.056*	
C11	0.17287 (16)	0.69097 (9)	0.16351 (7)	0.0214 (3)	
C12	0.09242 (18)	0.78138 (10)	0.14349 (9)	0.0307 (3)	
H12	0.0041	0.8008	0.1712	0.037*	
C13	0.1415 (2)	0.84243 (11)	0.08333 (9)	0.0403 (4)	
H13	0.0869	0.9037	0.0693	0.048*	
C14	0.2709 (2)	0.81376 (14)	0.04356 (11)	0.0477 (5)	
H14	0.3058	0.8562	0.0027	0.057*	
C15	0.3489 (2)	0.72443 (14)	0.06281 (10)	0.0463 (4)	
H15	0.4365	0.7049	0.0345	0.056*	
C16	0.30136 (18)	0.66239 (12)	0.12314 (9)	0.0317 (3)	
H16	0.3563	0.6010	0.1366	0.038*	
C21	0.20967 (14)	0.50274 (8)	0.26212 (8)	0.0197 (2)	
C22	0.26143 (16)	0.47526 (10)	0.33420 (8)	0.0253 (3)	
H22	0.2515	0.5230	0.3754	0.030*	
C23	0.32766 (18)	0.37759 (10)	0.34554 (9)	0.0318 (3)	
H23	0.3634	0.3582	0.3946	0.038*	
C24	0.34127 (18)	0.30872 (10)	0.28520 (10)	0.0326 (3)	
H24	0.3875	0.2422	0.2930	0.039*	
C25	0.28901 (17)	0.33492 (10)	0.21405 (9)	0.0308 (3)	
H25	0.2995	0.2867	0.1732	0.037*	
C26	0.22083 (16)	0.43177 (9)	0.20171 (8)	0.0249 (3)	
H26	0.1823	0.4495	0.1529	0.030*	
C31	−0.08483 (13)	0.59071 (9)	0.23979 (7)	0.0213 (2)	
C32	−0.17155 (16)	0.57888 (10)	0.30530 (8)	0.0273 (3)	
H32	−0.1306	0.5966	0.3536	0.033*	
C33	−0.31895 (18)	0.54080 (11)	0.29909 (10)	0.0350 (3)	
H33	−0.3800	0.5329	0.3433	0.042*	
C34	−0.37636 (17)	0.51460 (10)	0.22885 (10)	0.0370 (4)	
H34	−0.4768	0.4878	0.2251	0.044*	
C35	−0.29041 (18)	0.52654 (11)	0.16363 (10)	0.0344 (3)	
H35	−0.3321	0.5086	0.1155	0.041*	
C36	−0.14279 (16)	0.56485 (9)	0.16856 (9)	0.0278 (3)	
H36	−0.0826	0.5732	0.1241	0.033*	
Br1	0.449193 (19)	0.407894 (11)	0.011711 (8)	0.03538 (5)	
O90	0.3316 (2)	0.15581 (12)	0.02226 (11)	0.0685 (4)	
H901	0.2440 (19)	0.1498 (18)	0.0054 (14)	0.077 (9)*	
H902	0.340 (4)	0.2210 (13)	0.019 (2)	0.127 (13)*	
O91	0.0667 (2)	0.49128 (14)	0.01528 (11)	0.0693 (4)	
H911	0.017 (3)	0.4362 (16)	0.0119 (16)	0.091 (10)*	
H912	0.157 (2)	0.472 (2)	0.0104 (19)	0.117 (12)*	

### Atomic displacement parameters (Å^2^)

**Table:**

	*U*^11^	*U*^22^	*U*^33^	*U*^12^	*U*^13^	*U*^23^
P1	0.01657 (14)	0.01950 (12)	0.01802 (15)	−0.00030 (10)	0.00031 (14)	0.00029 (10)
C1	0.0313 (8)	0.0232 (5)	0.0218 (6)	−0.0020 (5)	−0.0001 (6)	−0.0033 (5)
C2	0.0371 (9)	0.0361 (7)	0.0379 (9)	−0.0122 (6)	−0.0038 (8)	−0.0069 (6)
C11	0.0222 (6)	0.0234 (5)	0.0184 (6)	−0.0038 (5)	−0.0015 (6)	0.0024 (4)
C12	0.0351 (8)	0.0284 (6)	0.0285 (7)	0.0013 (5)	−0.0018 (6)	0.0036 (5)
C13	0.0585 (11)	0.0302 (6)	0.0321 (8)	−0.0061 (7)	−0.0111 (9)	0.0098 (6)
C14	0.0624 (12)	0.0506 (9)	0.0302 (8)	−0.0207 (9)	0.0006 (9)	0.0158 (7)
C15	0.0424 (10)	0.0613 (10)	0.0354 (9)	−0.0063 (8)	0.0152 (9)	0.0080 (8)
C16	0.0286 (7)	0.0378 (7)	0.0289 (8)	0.0004 (6)	0.0058 (7)	0.0045 (6)
C21	0.0152 (6)	0.0197 (4)	0.0242 (7)	−0.0011 (4)	−0.0001 (6)	0.0020 (4)
C22	0.0251 (7)	0.0277 (6)	0.0231 (7)	−0.0003 (5)	−0.0004 (6)	0.0023 (5)
C23	0.0298 (7)	0.0326 (6)	0.0331 (8)	0.0015 (6)	−0.0036 (7)	0.0118 (5)
C24	0.0292 (7)	0.0206 (5)	0.0482 (9)	0.0023 (5)	0.0046 (8)	0.0064 (6)
C25	0.0318 (8)	0.0209 (5)	0.0396 (9)	−0.0028 (5)	0.0071 (7)	−0.0051 (6)
C26	0.0241 (7)	0.0256 (6)	0.0251 (7)	−0.0033 (5)	−0.0003 (6)	−0.0010 (5)
C31	0.0175 (5)	0.0195 (4)	0.0270 (6)	0.0003 (4)	0.0007 (5)	0.0004 (5)
C32	0.0223 (6)	0.0292 (6)	0.0304 (7)	0.0003 (5)	0.0010 (6)	0.0057 (5)
C33	0.0232 (7)	0.0343 (7)	0.0474 (10)	−0.0012 (6)	0.0075 (7)	0.0111 (6)
C34	0.0198 (7)	0.0282 (6)	0.0630 (11)	−0.0043 (5)	−0.0044 (8)	0.0072 (6)
C35	0.0267 (7)	0.0320 (6)	0.0444 (10)	−0.0018 (6)	−0.0106 (7)	−0.0053 (6)
C36	0.0228 (7)	0.0291 (6)	0.0315 (8)	−0.0004 (5)	−0.0023 (6)	−0.0025 (5)
Br1	0.03888 (9)	0.04024 (7)	0.02703 (7)	−0.00297 (6)	−0.00667 (7)	−0.00226 (5)
O90	0.0591 (10)	0.0589 (8)	0.0876 (13)	−0.0081 (7)	−0.0044 (10)	0.0171 (9)
O91	0.0564 (10)	0.0833 (10)	0.0680 (10)	0.0059 (9)	0.0105 (10)	−0.0101 (9)

### Geometric parameters (Å, °)

**Table:**

P1—C21	1.7896 (11)	C22—H22	0.9500
P1—C31	1.7913 (12)	C23—C24	1.382 (2)
P1—C11	1.7915 (13)	C23—H23	0.9500
P1—C1	1.7981 (13)	C24—C25	1.374 (2)
C1—C2	1.528 (2)	C24—H24	0.9500
C1—H1A	0.9900	C25—C26	1.3896 (18)
C1—H1B	0.9900	C25—H25	0.9500
C2—H2A	0.9800	C26—H26	0.9500
C2—H2B	0.9800	C31—C32	1.3896 (19)
C2—H2C	0.9800	C31—C36	1.3917 (19)
C11—C16	1.384 (2)	C32—C33	1.390 (2)
C11—C12	1.3976 (18)	C32—H32	0.9500
C12—C13	1.382 (2)	C33—C34	1.375 (2)
C12—H12	0.9500	C33—H33	0.9500
C13—C14	1.385 (3)	C34—C35	1.382 (2)
C13—H13	0.9500	C34—H34	0.9500
C14—C15	1.372 (3)	C35—C36	1.391 (2)
C14—H14	0.9500	C35—H35	0.9500
C15—C16	1.388 (2)	C36—H36	0.9500
C15—H15	0.9500	O90—H901	0.830 (15)
C16—H16	0.9500	O90—H902	0.836 (15)
C21—C22	1.3914 (19)	O91—H911	0.830 (15)
C21—C26	1.3983 (18)	O91—H912	0.836 (16)
C22—C23	1.3889 (18)		
			
C21—P1—C31	105.57 (5)	C22—C21—P1	120.13 (10)
C21—P1—C11	112.48 (6)	C26—C21—P1	119.22 (10)
C31—P1—C11	109.65 (6)	C23—C22—C21	119.58 (13)
C21—P1—C1	110.27 (6)	C23—C22—H22	120.2
C31—P1—C1	111.65 (6)	C21—C22—H22	120.2
C11—P1—C1	107.28 (6)	C24—C23—C22	119.71 (14)
C2—C1—P1	111.91 (10)	C24—C23—H23	120.1
C2—C1—H1A	109.2	C22—C23—H23	120.1
P1—C1—H1A	109.2	C25—C24—C23	121.01 (12)
C2—C1—H1B	109.2	C25—C24—H24	119.5
P1—C1—H1B	109.2	C23—C24—H24	119.5
H1A—C1—H1B	107.9	C24—C25—C26	120.18 (13)
C1—C2—H2A	109.5	C24—C25—H25	119.9
C1—C2—H2B	109.5	C26—C25—H25	119.9
H2A—C2—H2B	109.5	C25—C26—C21	119.11 (13)
C1—C2—H2C	109.5	C25—C26—H26	120.4
H2A—C2—H2C	109.5	C21—C26—H26	120.4
H2B—C2—H2C	109.5	C32—C31—C36	121.22 (12)
C16—C11—C12	120.13 (12)	C32—C31—P1	119.43 (10)
C16—C11—P1	122.97 (10)	C36—C31—P1	118.90 (10)
C12—C11—P1	116.61 (11)	C33—C32—C31	119.07 (14)
C13—C12—C11	119.88 (14)	C33—C32—H32	120.5
C13—C12—H12	120.1	C31—C32—H32	120.5
C11—C12—H12	120.1	C34—C33—C32	119.89 (15)
C12—C13—C14	119.65 (15)	C34—C33—H33	120.1
C12—C13—H13	120.2	C32—C33—H33	120.1
C14—C13—H13	120.2	C33—C34—C35	121.12 (13)
C15—C14—C13	120.40 (15)	C33—C34—H34	119.4
C15—C14—H14	119.8	C35—C34—H34	119.4
C13—C14—H14	119.8	C34—C35—C36	119.90 (15)
C14—C15—C16	120.67 (17)	C34—C35—H35	120.0
C14—C15—H15	119.7	C36—C35—H35	120.0
C16—C15—H15	119.7	C35—C36—C31	118.80 (14)
C11—C16—C15	119.25 (15)	C35—C36—H36	120.6
C11—C16—H16	120.4	C31—C36—H36	120.6
C15—C16—H16	120.4	H901—O90—H902	98.5 (19)
C22—C21—C26	120.38 (11)	H911—O91—H912	104 (2)
			
C21—P1—C1—C2	64.80 (11)	C26—C21—C22—C23	1.5 (2)
C31—P1—C1—C2	−178.17 (10)	P1—C21—C22—C23	175.58 (11)
C11—P1—C1—C2	−58.01 (12)	C21—C22—C23—C24	−0.1 (2)
C21—P1—C11—C16	−12.47 (14)	C22—C23—C24—C25	−0.6 (2)
C31—P1—C11—C16	−129.61 (12)	C23—C24—C25—C26	−0.2 (2)
C1—P1—C11—C16	108.96 (12)	C24—C25—C26—C21	1.5 (2)
C21—P1—C11—C12	173.72 (10)	C22—C21—C26—C25	−2.20 (19)
C31—P1—C11—C12	56.58 (12)	P1—C21—C26—C25	−176.35 (10)
C1—P1—C11—C12	−64.85 (12)	C21—P1—C31—C32	84.70 (11)
C16—C11—C12—C13	−0.1 (2)	C11—P1—C31—C32	−153.90 (10)
P1—C11—C12—C13	173.87 (12)	C1—P1—C31—C32	−35.14 (12)
C11—C12—C13—C14	−0.4 (2)	C21—P1—C31—C36	−87.72 (11)
C12—C13—C14—C15	1.0 (3)	C11—P1—C31—C36	33.68 (11)
C13—C14—C15—C16	−1.1 (3)	C1—P1—C31—C36	152.44 (10)
C12—C11—C16—C15	0.0 (2)	C36—C31—C32—C33	−0.21 (19)
P1—C11—C16—C15	−173.60 (13)	P1—C31—C32—C33	−172.45 (10)
C14—C15—C16—C11	0.6 (3)	C31—C32—C33—C34	0.6 (2)
C31—P1—C21—C22	−103.52 (11)	C32—C33—C34—C35	−0.7 (2)
C11—P1—C21—C22	136.94 (11)	C33—C34—C35—C36	0.5 (2)
C1—P1—C21—C22	17.23 (12)	C34—C35—C36—C31	−0.2 (2)
C31—P1—C21—C26	70.65 (11)	C32—C31—C36—C35	0.01 (19)
C11—P1—C21—C26	−48.89 (12)	P1—C31—C36—C35	172.29 (10)
C1—P1—C21—C26	−168.60 (10)		

### Hydrogen-bond geometry (Å, °)

**Table:**

*D*—H···*A*	*D*—H	H···*A*	*D*···*A*	*D*—H···*A*
O90—H901···Br1^i^	0.83 (2)	2.71 (2)	3.5137 (18)	162 (2)
O90—H902···Br1	0.84 (2)	2.57 (2)	3.3806 (15)	163 (3)
O91—H911···O90^i^	0.83 (2)	2.10 (2)	2.869 (3)	155 (3)
O91—H912···Br1	0.84 (2)	2.70 (2)	3.5315 (17)	174 (3)
C35—H35···Br1^ii^	0.95	2.95	3.8305 (16)	155
C1—H1B···Br1^iii^	0.99	2.70	3.6843 (14)	174

Symmetry codes: (i) *x*−1/2, −*y*+1/2, −*z*; (ii) *x*−1, *y*, *z*; (iii) −*x*+1/2, −*y*+1, *z*+1/2.
